# Ontogeny of OPN4, OPN5, GnRH and GnIH mRNA Expression in the Posthatch Male and Female Pekin Duck (*Anas platyrhynchos domesticus*) Suggests OPN4 May Have Additional Functions beyond Reproduction

**DOI:** 10.3390/ani11041121

**Published:** 2021-04-14

**Authors:** Brooke Van Wyk, Gregory Fraley

**Affiliations:** 1 Biology Department, Hope College, Holland, MI 49423, USA; brooke.vanwyk@hope.edu; 2 Department of Animal Sciences, Purdue University, West Lafayette, IN 47907, USA

**Keywords:** melanopsin, puberty, neonate, photoreception

## Abstract

**Simple Summary:**

Birds perceive light differently than mammals. Unlike mammals, birds’ retinas do not participate in the non-image forming, seasonal breeding aspects of light perception. Birds have deep brain photoreceptors that are involved with the seasonal increase in fertility. Until this study, it was unknown how these brain photoreceptors developed after hatching. Our novel finding is that at least one of the photoreceptors, OPN4, is expressed at high levels on the day of hatching, which suggests that this photoreceptor has functions beyond just the reproductive system. These findings could change how we light poultry barns to improve the growth, health and welfare of our poultry species.

**Abstract:**

The hypothalamic–pituitary–gonadal axis (HPG) is known to be regulated by daylength through the deep brain photoreceptor (DBP) system. The post-hatch ontogeny is not known for any of the DBPs. We set out to determine the ontogeny of OPN4 and OPN5 gene expression relative to GnRH and GnIH using qRT-PCR. Brains and serum were collected from five drakes and five hens on the day of hatching (Day 0) and again at 2, 4, 6, 10, 14, 19, 25 and 31 weeks of age and analyzed by qRT-PCR. Hen and drake serum was assayed for circulating levels of estradiol and testosterone, respectively. Data were analyzed between sexes over time using a repeated measures two-way ANOVA. Interestingly, the results show that on the day of hatching (Day 0), ducks showed adult-like levels of relative OPN4, but not OPN5, gene expression. During week 10, DBP levels increased, achieving highest relative expression levels at week 19 that maintained through week 31, typically peak fertility in ducks. GnRH mRNA levels increased following the DBP expression at the onset of puberty, and gonadal steroids increased after GnRH at week 14 while estradiol preceded testosterone. GnIH mRNA levels did not appreciably change during the time course of this experiment. These observations suggest that OPN4 may be active during the peri-hatch period and may have physiological roles beyond puberty and fertility.

## 1. Introduction

Lighting systems in commercial poultry have come under increased scrutiny over the last decade. Long daylengths are necessary in order to maintain reproduction in all poultry species, and the duck is no exception [1,2,3]. Previous studies have attempted to understand how types of lighting, intensity of light, and color spectra of light are critical to maximize the reproduction and welfare of both meat and breeder ducks [4,5,6,7]. As with all of our poultry species, ducks are seasonal breeders and as such require a minimum of 14 h of light per day in order to maintain fertility [8]. Despite being maintained on a long daylength, there are still inexplicable drops in fertility during winter months, along with increased unwanted behaviors, such as feather picking, aggression, and mislaid eggs [9,10,11]. In the large part, we do not understand these seasonal phenomena because there is a dearth of information in the literature about how ducks—or other birds—perceive light, not only visually but for seasonal reproduction. However, a series of deep brain photoreceptors (DBPs) have been described in the diencephalon that are involved with maintaining fertility in response to daylength in poultry. 

DBPs activate the hypothalamic–pituitary–gonadal axis (HPG) through a neuroendocrine cascade that involves thyroid hormone. At least four DBPs have been putatively identified in the duck, including opsin 4 and opsin 5 [7,12]. It has been shown that DBPs stimulate neurons that signal the mediobasal hypothalamus (MBH), which is used in photoperiodic time measurement. Upon stimulation, the MBH produces the type 2 deiodinase (DIO2) that converts thyoxine (T4) to triiodothyronine (T3). Conversely, during short day lengths, the MBH produces a thyroid hormone (TH)-deactivating enzyme from the DIO3 gene, which converts T4 and T3 into inactive reverse triiodothyronine, an inactive form of thyroid hormone [13]. Thyroid hormone is critical for many physiological changes associated with post-hatch development and maturation of the central nervous system (CNS). Thyroid hormone is also necessary for normal growth and CNS development, as well as the onset of puberty [14]. Puberty is defined as the initial increase in GnRH secretion [15,16]. However, the temporal ontogeny of DBPs relative to hypothalamic maturation of the HPG (GnRH and GnIH) is currently unknown in any avian species. The normal development of the CNS and sexual maturation of GnRH could be driven by differential temporal expression among the DBPs. 

The purpose of our study was to determine the temporal expression of opsin 4 and opsin 5 as they relate to the ontogeny of GnRH and GnIH gene expression. In order to accomplish this, we euthanized male and female ducks from hatch through the age associated with peak fertility. A more thorough understanding of the expression pattern of DBPs and their respective roles prior to puberty could enable us to better understand the physiological role of light beyond just reproduction, and ultimately to design lighting systems to utilize the temporal expression of each individual DBP in order to maximize the reproductive performance, production, and welfare of Pekin ducks. 

## 2. Materials and Methods

### 2.1. Animals

Brains from 54 ducklings/ducks were collected at Maple Leaf Farms (Leesburg, IN, USA) from breeder stock housed in commercial barns that produces Gx strain of meat birds. Three male and three female birds were randomly selected on the day of hatch (week 0), and again on weeks 2, 4, 6, 10, 14, 19, 25, and 31. All ducks were raised following industry standards for heat, water, and feed. Further, all ducks were raised under fluorescent, J-lamps, which emit a full spectrum of visible light ([17]; ~5000 kelvin, ~65 lux/25.74 uW/cm^2^), with lights on at 0300 h (LD 18:6). Visual signs of puberty, as defined by age at first lay, typically occur reliably between 15 and 16 weeks of age [9]. The onset of puberty is defined here as the initial rise in GnRH mRNA (for review see [18]). All samples were collected between 0600 and 0800 h each day. Procedures were approved by Hope College’s IACUC (#011803R). 

### 2.2. Tissue and RNA Collection

Brains were removed from the ducks, diencephali (thalamus and hypothalamus) dissected, and immediately frozen on dry ice for transportation. Diencephali were dissected rostrally at the septomesencephalic tract, caudally at the third cranial nerve, and dorsally ~5 mm above the anterior commissure. Diencephali were stored at −80 °C until being processed. RNA was isolated from the diencephali using commercially available columns following the manufacturer’s recommendations (kit #75162, Qiagen, Germantown, MD, USA). RNA was stored at −80 °C until being used for qRT-PCR. Prior to euthanasia, blood was collected from the tibial vein and placed into serum separator tubes, centrifuged, and the serum was stored at −20 °C until being analyzed for circulating testosterone (T) or estradiol (E_2_) in drakes and hens, respectively. 

### 2.3. qRT-PCR

To complete qRT-PCR analyses, we utilized a Superscript VILO Invitrogen (Carlsbad, CA, USA) cDNA synthesis kit. Single-stranded cDNA was synthesized from 2 μg total cellular RNA using oligo(dT)16 primer and superscript II Reverse Transcriptase (Gibco BRL, Invitrogen Corp., Carlsbad, CA, USA), as recommended by the manufacturer. Five micrograms of RNA were used to perform a reverse transcription reaction using second round primers, and then second strand was synthesized using oligo(dT) primer. The 3′ end specific oligonucleotide primers were designed within 300 bp from the 3′ end of the transcript and used in qRT-PCR for each of the DPBs or neuropeptides (see Table 1). Developmental reference genes (GAPDH and β-actin) were averaged and used at every time point for all gene expression analyses. The amplification profile of β-actin and GAPDH, DBPs and the neuropeptides consisted of 36 cycles each for 1 min at 95 °C, 30 s at 54 °C, and 1 min at 72 °C, respectively. The cycles were previously determined to be within the linear range. Final qRT-PCR was performed using the iTaq SYBR Green Supermix (BioRad Inc.; Hercules, CA, USA) following the manufacturers recommendation using a CFX96 Touch REalt Time System (BioRad Inc.; Hercules, CA, USA). Fold changes were determined following by first averaging the ct values for all samples. The delta ct was determined by taking the gene of interest average minus the β-actin average. The delta-delta ct was determined by subtracting the treatment delta ct value from the control delta ct value; fold-change was calculated by using the delta-delta ct value (x) and calculating 2^−x^. 

### 2.4. Hormone Analyses

T and E_2_ were analyzed by ELISA (Caymen Chemical, #582701 and # 501890, respectively; Ann Arbor, MI, USA). In order to validate the kit for use in ducks, 20 mL of blood was collected from drakes and hens and charcoal-stripped with 2 g of activated charcoal (Sigma Aldrich, St. Louis, MO, USA) on a shaker table for 2 h. The samples were centrifuged (4000 RPM, 15 min at room temp.) and the steroid-free serum was removed from the charcoal pellet. The steroid-free serum was then used to produce a standard curve following the manufacturer’s suggestions. For each kit, a standard curve was run using the kit’s buffer and compared to the respective standard curve produced with the steroid-free serum. Buffer vs. serum standard curves were very similar for each kit (R^2^ > 0.98). A pregnenolone standard curve made with either buffer or steroid-free serum produced no measurable binding suggesting that either kit would not cross-react with pregnenalone. Finally, 6 sets of triplicates of the charcoal stripped serum produced no measurable binding, indicating that the sample was steroid-free. The E_2_ kit’s assay range is 0.61–10,000 pg/mL and sensitivity is 20 pg/mL. Hen samples were run undiluted in duplicate following manufacturers recommendation with a final incubation time of 60 min. The T kit assay range was 3.9–500 pg/mL and sensitivity of 6 pg/mL. Drake samples were diluted 1:4 in the kit’s assay buffer and run in duplicate following the manufacturer’s recommendation with a final incubation time of 90 min to achieve B_0_ = 0.3. All plates were read using a Synergy Lx (Biotek, Inc. Winooski, VT 05404, USA) at 405 (T) or 414 nm (E_2_).

### 2.5. Statistical Analyses

Relative mRNA levels were analyzed ad hoc using a 2-way ANOVA at each age using Mac JMP (JMP 9; SAS Institute, Raleigh, NC, USA) followed by a Fisher’s PLSD post hoc test to determine differences between pairs of treatment groups. Endocrine data were analyzed by a 1-way ANOVA for each hormone. A *p* value < 0.05 was considered significant.

## 3. Results

**Figure 1:**
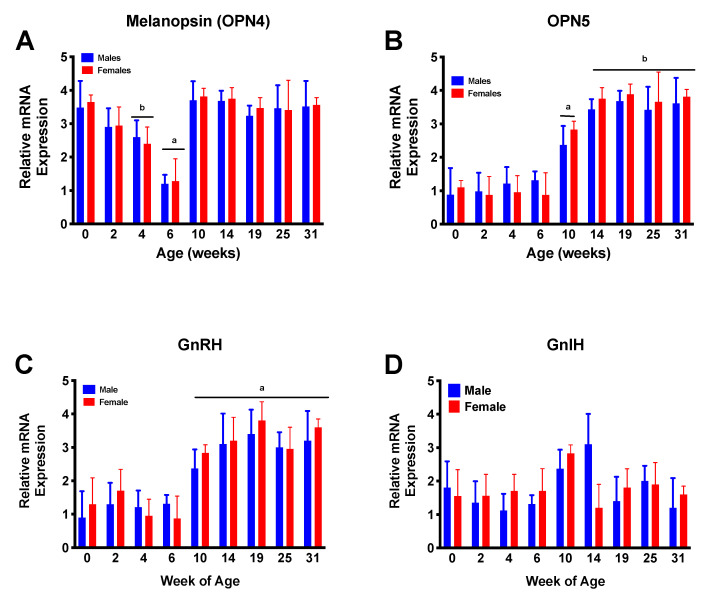
Ontogeny of relative mRNA expression of opsin 4 (OPN4; (**A**)), opsin 5 (OPN5; (**B**)), gonadotropin releasing hormone (GnRH; (**C**)), and gonadotropin inhibitory hormone (GnIH; (**D**)). Relative OPN4 expression was high just after hatch then dropped over the first 6 weeks of life, then once again increased prior to the increase in relative GnRH mRNA expression. OPN5 expression remained low until prior to the increase in GnRH expression. No differences were observed in GnIH relative mRNA expression at any age. Letters indicate statistically different groups at *p* < 0.05.

Ad hoc analyses revealed no sex differences in mRNA expression for any of the neuroendocrine elements. However, a significant (*p* < 0.001) age effect was noted in the expression of OPN4, OPN5, and GnRH, but not GnIH, during post-hatch development. On the day of hatch, male and female ducklings showed near-adult levels of OPN4 mRNA. By week 4, these dropped off to baseline, then increased again after 10 weeks of age. OPN5 mRNA, on the other hand, remained at baseline levels from the day of hatch until week 10 and achieved adult-like (as represented by peak fertility at ~25 weeks) levels by week 14. GnRH mRNA levels remained constant from hatch until just prior to week 10, at which time they increased to adult levels. GnIH mRNA levels did not change appreciably at any age during this study. Figure 1 illustrates these results.

**Figure 2:**
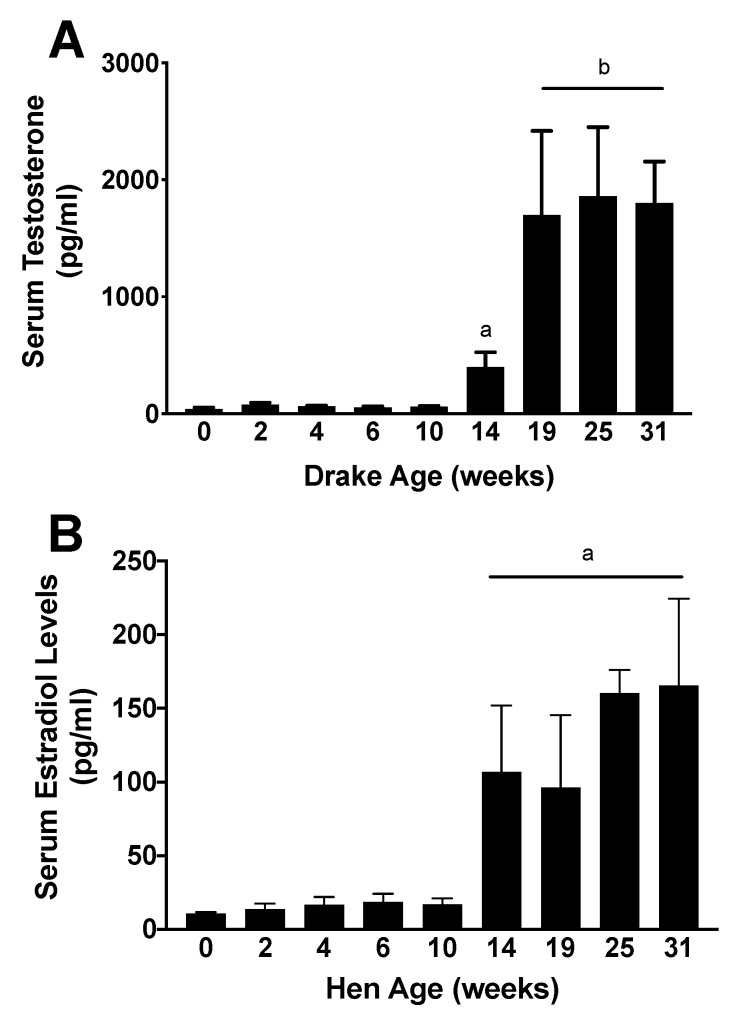
Serum T and E_2_ concentrations in drakes and hens, respectively. (**A**) Serum T concentrations in drakes are at minimal concentrations from hatch through 10 weeks of age. T concentrations were significantly greater by 14 weeks of age, and again significantly increased 19 weeks and maintained into adulthood. Letters indicate statistically significant groups, a = *p* < 0.05, b = *p* < 0.01. (**B**) Serum E_2_ concentrations were also at baseline from hatch through week 10 and were significantly increased to adult-like concentrations by week 19. a = *p* < 0.001. Data suggest that gonadal development is occurring by week 14 and may be more advanced in hens compared to drakes.

First eggs were observed in this flock at week 16, and peak fertility, as determined by <15% clear eggs at candling, occurred by week 25 (data not shown due to commercial housing that makes it impossible to trace a given egg to a specific hen). Hormone analyses showed that both T and E_2_ concentrations were minimal from hatch through week 10 in drakes and hens, respectively. By week 14, E_2_ concentrations were significantly (*p* < 0.001) increased to adult levels in hens. However, drakes’ T concentrations began to significantly increase at week 14 (*p* < 0.05) but did not reach adult-like concentrations until week 19 (*p* < 0.01). The hormone data are presented in Figure 2.

## 4. Discussion

Thus far, deep brain photoreceptors have been studied exclusively in adult avian species, and little is known about the temporal development of DBPs. In order to determine the developmental expression of DBPs in Pekin ducks, diencephali from specific age groups were homogenized, and the RNA was extracted from that tissue. Using that RNA, qPCR was used to determine expression levels of genes encoding the DBPs for OPN5, OPN4, GnRH and GnIH. It was expected that DBP expression would increase prior to the onset of puberty, and this expectation was confirmed as the levels of both DBPs increased prior to the increase in GnRH. Although this study is not able to elicit causative relationships among the various mRNAs, it is interesting to point out the relative temporal expression around the onset of puberty (defined as the initial rise in GnRH mRNA levels [15,16]). There is an apparent initial increase in OPN4, followed by OPN5, then GnRH prior to the increase in gonadal steroids and onset of lay (16 weeks), followed by peak fertility (25 weeks). The slightly earlier timing of E_2_ compared to T is somewhat in agreement with the initial onset of lay in the flock at 16 weeks, and peak fertility at 25 weeks. The relative temporal roles of each of these hypothalamic moieties has been described in other avian species [19,20,21,22]. Our most important finding, however, is that OPN4 was expressed at near-adult levels on the day of hatching, dropped to baseline until week 10 when levels again increased, at least 4 weeks earlier than the increased gonadal function as evidenced by gonadal steroid output. OPN5 remained at baseline levels until week 10, after which it also increased to adult levels by week 14. Surprisingly, no sex differences were observed during the ontogeny of DBPs. Perhaps a lighting paradigm could be developed to maximize production and welfare of birds based on the expression pattern and peak excitation energy of different DBPs.

A potential new lighting paradigm would function by taking advantage of the wavelength-specificity of different DBPs. Although the effects of monochromatic light on fertility and behavior are quite species-specific, previous studies from our lab and others have indicated that monochromatic red light may have a “calming” effect on ducks [5,6,10], and that blue light increases activity, reduces fertility and may produce stress [5,6,10,23,24]. In order to maximize fertility in Pekin ducks, a combination of both red and blue light is required [10,12]. These observations are apparently contradictory given the peak excitation potential for OPN4 and OPN5. OPN5 is responsive to short wavelengths, with peak excitation at 420 nm [25]. Additionally, short wavelength light induced photoperiodic responses in quail when other photoreceptors had been eliminated [25]. Opsin 4 is maximally stimulated by light around 480 nm [26]. Studies in the duck have shown that elimination of OPN4 expression in the brain of drakes causes gonadal regression [27]. However, previous studies in galliforms and songbirds have suggested that longer wavelengths of light penetrate brain tissue best and will stimulate gonadal recrudescence [28,29,30,31,32]. These conflicting findings emphasize the importance for future studies to better understand how the properties of light change as it passes through feathers and tissue for brain photoreception. If we can better understand the temporal expression, peak excitation energies, and physics of the light properties as it passes through tissues, then perhaps we could reverse engineer a lighting system that could reduce unwanted behaviors such as feather picking, aggression, and mislaid eggs, and prevent seasonal losses in fertility [10,17].

The brains of birds receive information about light via different mechanisms than mammals. Mammalian photoreceptivity includes retinal image and non-image forming cells. Bilateral enucleation in birds does not affect seasonal fertility or the HPG axis [33]. Gonads will either regress, or will not recrudesce, when light is prevented from penetrating the skull [30,34,35,36]. Thus, the DBPs have been studied as a mechanism for photoreceptivity in birds (reviewed in [37]). The activation and deactivation of DPBs have been linked to recrudescence and regression, respectively, including in the duck [12,17,20,38,39,40]. Furthermore, we have previously shown that insufficient light intensity will also decrease fertility and GnRH mRNA expression levels, a physiological function that is likely linked to reduced serum thyroid levels [7]. However, the increased OPN4 mRNA expression levels at hatching compared to OPN5, GnRH and GnIH mRNAs, and gonadal steroid levels, suggest that OPN4 may have other functions beyond reproduction, but perhaps still linked to the well-established relationship with the thyroid axis [19,37,41,42,43,44,45,46]. We have previously demonstrated that there are seasonal losses in fertility and an increase in mislaid eggs [7,17]. Perhaps a better understanding of early post-hatch expression of DBPs could enable us to better design lighting systems to prevent these two production and welfare issues, respectively. Early physiological functions of DBPs could involve their established relationship with thyroid hormones.

Thyroid hormones in birds have numerous functions including weight gain, fattening, and muscle hypertrophy to prepare birds for migration [47]. Type 2 iodothyronine deiodinase (DIO2), which converts T_4_ to T_3_ in birds [37,45,48], is induced by photostimulation [49]. DIO2 activity is increased specifically in the diencephalon [49] during long day stimulation compared to short days. Activation of the hypothalamic-pituitary-thyroid axis has long been suggested as a necessary component of DBP function [19,37,41,42,43,44,45,46]; however, this has not been directly confirmed in the duck. Thyroid hormones are closely related to the hatching process, particularly in precocial species such as ducks. The necessity of thyroid hormone secretion for normal development of the central nervous system has been well established for nearly a century. It may be possible that activation of OPN4 at hatch leads to the increase in thyroid activity to aide in the development of the CNS; however, this cannot be ascertained from this study alone. Sex differences in post-hatch thyroid hormone levels have been shown in zebra finches, however not in precocial species [50], which is somewhat in agreement with the lack of sex differences that we observed in this experiment in DBP expression. However, a large peri-hatch increase in hypothalamic–pituitary–thyroid axis activity is seen in precocial, but not altricial species [51]. Increased thyroid hormone levels around hatch have been associated with the critical period of post-hatch imprinting, and exogenous T_3_ at this time can enhance imprinting and learning in chicks [52]. Although the relationship of peri-hatch thyroid hormones and imprinting and learning in Pekin ducks is unknown, it has been demonstrated that increased thyroid hormones near the end of incubation are involved in cholinergic- and adrenergic-mediated regulation of cardiovascular development [53,54], as well as the initial thermoregulatory responses to cooling at hatch, and ultimately thermoregulation [55,56,57]. Thus, the potential link between DBP expression and the hypothalamic-pituitary-thyroid system may have important impacts upon the behavior, welfare and physiological function of our domestic poultry species beyond the reproductive system. The purpose of this experiment was to highlight expression patterns of OPN4 and OPN5, however, a determination of the physiological role of these expression patterns is beyond the scope of this study.

Seasonal variations have been observed in GnRH neuronal activity, and prior to puberty in birds [58]. GnRH mRNA expression is increased during the breeding season and is decreased during gonadal regression [59,60]. Gonadal regression can be induced by numerous factors, including an increased hypothalamic GnIH activity [61,62]. In birds, GnIH is thought to inhibit gonadotropin release and sexual behavior [62,63,64], and stimulates feeding in numerous avian species, [65,66,67] and the duck is no exception [62]. However, previous studies from our lab have also revealed few to no GnIH mRNA changes associated with changes in light or gonadal status [12]. It is possible that our current study had insufficiently small time increments in order to resolve small changes in DBP or GnIH mRNAs; previous studies have shown that gonadal development requires the interplay of numerous DBPs [12,19,27,68]. It is likely that DBP stimulation does not lead to the activation or deactivation of GnIH neurons. Little evidence in the duck suggests that GnIH plays a role in pubertal timing [62]. However, growing evidence in other avian species suggests that it may provide a regulatory mechanism for HPG function during times of stress [14,69,70]. It is likely that the primary role of this neuropeptide has yet to be elucidated.

## 5. Conclusions

Melanopsin (OPN4), but not OPN5, mRNA is expressed at near adult-like levels in both the hen and drake Pekin duckling at hatch. The neonatal expression of this DBP suggests that it may have further physiological roles beyond reproduction.

## Figures and Tables

**Table 1:** qRT-PCR primer sequences.

Target	Gene	Forward	Reverse
DBP	OPN4	CTCGCCATAGAACATCCGCA	ACTGAACAGGCTACTCCCCTT
	OPN5	TTT CTC ACC GCT GGA TCT TT	CAG GCA GAT AAA GGC ATG GTG T
Reproductive	GnRH-1	ATC GCA AAC GAA ATG GAA AG	CTG GCT TCT CCT TCG ATC AG
	GnIH	TAA CAC CGC ATG GTA TGT GC	CTC CTC TGC TCT TCC TCC AA
Housekeeping	GAPDH	GGTTGTCTCCTGCGACTTCA	TCCTTGGATGCCATGTGGAC
	β-actin	CAC AAT GTA CCC GGG CAT CG	ACA TCT GCT GGA AGG TGG AC

## Data Availability

The data presented in this study are available on request from the corresponding author.
